# Postural orthostatic tachycardia syndrome explained using a baroreflex response model

**DOI:** 10.1098/rsif.2022.0220

**Published:** 2022-08-24

**Authors:** Justen R. Geddes, Johnny T. Ottesen, Jesper Mehlsen, Mette S. Olufsen

**Affiliations:** ^1^ Department of Mathematics, North Carolina State University, Raleigh, NC 27695, USA; ^2^ Department of Science and Environment and Centre for Mathematical Modeling – Human Health and Disease, Roskilde University, Roskilde, Denmark; ^3^ Section for Surgical Pathophysiology, Rigshospitalet, Copenhagen, Denmark

**Keywords:** POTS, baroreflex, autonomic dysfunction, head-up tilt, mathematical modelling

## Abstract

Patients with postural orthostatic tachycardia syndrome (POTS) experience an excessive increase in heart rate (HR) and low-frequency (∼0.1 Hz) blood pressure (BP) and HR oscillations upon head-up tilt (HUT). These responses are attributed to increased baroreflex (BR) responses modulating sympathetic and parasympathetic signalling. This study uses a closed-loop cardiovascular compartment model controlled by the BR to predict BP and HR dynamics in response to HUT. The cardiovascular model predicts these quantities in the left ventricle, upper and lower body arteries and veins. HUT is simulated by letting gravity shift blood volume (BV) from the upper to the lower body compartments, and the BR control is modelled using set-point functions modulating peripheral vascular resistance, compliance, and cardiac contractility in response to changes in mean carotid BP. We demonstrate that modulation of parameters characterizing BR sensitivity allows us to predict the persistent increase in HR and the low-frequency BP and HR oscillations observed in POTS patients. Moreover, by increasing BR sensitivity, inhibiting BR control of the lower body vasculature, and decreasing central BV, we demonstrate that it is possible to simulate patients with neuropathic and hyperadrenergic POTS.

## 1. Introduction

Postural orthostatic tachycardia syndrome (POTS) is a form of autonomic dysfunction characterized by an excessive increase in heart rate (HR) (tachycardia) upon transitioning from a supine to an upright position in the absence of orthostatic hypotension and other conditions provoking sinus tachycardia. A positive diagnosis also requires a history of persistent (at least six months) symptoms, including brain fog, palpitations, visual blurring or dizziness, [1–3]. Since POTS is a phenotype of autonomic dysfunction and not a specific disease, it is difficult to identify the compromised mechanisms. This is partly due to POTS’ numerous potential pathologies, including neuropathy or the presence of agonistic antibodies binding to specific adrenergic receptors [3,4]. POTS is typically diagnosed by examining beat-to-beat HR, and blood pressure (BP) signals measured during a postural challenge, such as head-up tilt (HUT) or active standing [5]. These signals, measured continuously, are reported along with a description of symptoms, yet diagnosis primarily relies on a single quantity—tachycardia (HR increase ≥ 30 bpm, ≥ 40 bpm for adolescents) [5,6].

Diagnosis of POTS from postural tachycardia is problematic as the single measure does not provide adequate insight into the syndrome’s underlying causes. Recent studies [7–9] have reported that POTS patients exhibit tachycardia (increasing HR more than normal) in response to postural change. They also experience increased ∼0.1 Hz HR and BP oscillations. In our previous study [7] examining data from 28 controls and 28 POTS patients, we found that ∼0.1 Hz oscillations had a higher amplitude during HUT and that phase difference is smaller in POTS patients at rest and during HUT. These observations agree with Stewart *et al.* [8,9] reporting increased ∼0.1 Hz oscillations in cerebral blood flow during HUT. Adding a marker characterizing low-frequency oscillation may improve diagnoses, but it should be accompanied by better characteristics describing what causes this feature to change.

Moreover, POTS has several phenotypes [1,3,10,11] including (1) neuropathic POTS caused by neuropathy in the vascular beds, particularly in the lower body; (2) hypovolaemic POTS attributed to low fluid volume, and (3) hyperadrenergic POTS characterized by high levels of circulating norepinephrine during postural change inducing an exaggerated sympathetic response.

It is known that POTS is associated with modulation of the baroreflex (BR) function [3,7]. Several hypotheses have been put forward suggesting what parts of the system may be compromised, though it is difficult to determine how each factor impacts dynamics. As a result, patients receive a series of tests to examine their dynamic response, but more methods are needed to examine the output. This study uses mathematical modelling to investigate how the system responds when parameters associated with BR sensitivity are varied and if modulation can differentiate POTS phenotypes. Results provide new insight, which has the potential to be incorporated into diagnostic criteria providing a more elaborate diagnosis.

For healthy people, the BR system operates via negative feedback modulating sympathetic and parasympathetic nerve activity, mitigating BP changes. During HUT, stretch receptors in the carotid sinus detect changes in BP modulating firing rate in the glossopharyngeal nerve, which sends signals to the nucleus tractus solitarius (NTS). From here, the signals are transmitted via the efferent sympathetic and parasympathetic nerves. HR is modulated by changes in sympathetic and parasympathetic nerve firing. The parasympathetic nerves primarily modulate heart rate, while the sympathetic nervous system modulates peripheral vascular resistance and cardiac contractility. At rest, sympathetic activity is low (20% of its maximum), while the parasympathetic activity is high (80% of its maximum) [12]. In response to a decrease in BP, the afferent signalling is inhibited, leading to parasympathetic withdrawal and sympathetic stimulation, which increase HR, cardiac contractility and peripheral resistance [13]. Numerous studies have examined BR signalling [9,14,15], and it has been established that BP and HR are controlled by negative feedback with a resonance frequency of ∼0.1 Hz. This response is easily distinguished from HR (with a frequency of ∼1 Hz) and respiration (which oscillates with a frequency of 0.2−0.3 Hz) [15].

Several recent studies have examined the magnitude and phase of the low-frequency (∼0.1 Hz) BP and HR oscillations in POTS patients [7–9]. The studies by Stewart *et al.* [8], and Medow *et al.* [9] used transcranial Doppler measurements of cerebral blood flow and finger arterial plethysmography to analyse blood flow and HR oscillations in response to a postural challenge. Using auto-spectral and transfer function analysis, they reported that increased low-frequency oscillations in arterial pressure lead to increased oscillations in cerebral blood flow, which they suggest may be responsible for the ‘brain fog’ experienced by many POTS patients. These results agree with our empirical mode decomposition findings examining BP and HR signals measured during HUT from female patients diagnosed with POTS. However, as noted in our previous study [7] not distinguishing POTS phenotypes, low-frequency oscillations are increased after HUT in all POTS patients, but there is significant variation among individuals. This suggests that oscillation amplitude and phase difference may differ among the POTS phenotypes. In summary, from previous work [7] and other previous studies [1,3,11], it is clear that the BR is compromised. Still, more work is needed to explain how specific pathophysiology impacts HR and BP dynamics.

To model the BR response to HUT, additional considerations are needed. First, the cardiovascular model must be adapted to account for the gravitational pooling of blood in the lower extremities. Second, the BR model must be adjusted to account for the orthostatic stress challenge. Several studies have examined this phenomenon, e.g. [16–22]. The model by Olufsen *et al.* [23] used HR as an input to predict BP during active standing for a healthy young adult, estimating patient-specific parameters modulating peripheral vascular resistance and vascular compliance. Williams *et al.* [21] adapted this approach to study the response to HUT. Matzuka *et al.* [22] used Kalman filtering and Williams *et al.* [24] used optimal control to estimate model parameters.

While these studies captured variations in response to a postural change, to our knowledge, only a few studies have attempted to test if dynamical systems models display ∼0.1 Hz oscillations. The study by Heldt *et al.* [25] built a model predicting low-frequency oscillations in astronauts undergoing an active standing test using a BR control model. They found that the low-frequency oscillations emerge but do not persist after the transition from sitting to standing. Another attempt was made by Hammer & Saul [26], who used an open- and closed-loop BR model to predict postural change. This model uses arterial BP as an input to predict HR. While this model examines the ∼0.1 Hz oscillations, it does not study how the response changes in time; instead, it quantifies stability at fixed operating points responsible for low-frequency oscillations. More recently, Ishbulatov *et al.* [27] used a closed-loop BR model to replicate low-frequency aspects of patient data during a passive HUT test. This study analyses how a healthy human body adapts to an orthostatic challenge. However, this model is complex and does not study the response in POTS patients.

To our knowledge, no previous studies have combined a mechanistic model with signal analysis to explain the emergence and modulation of the low-frequency oscillations for POTS patients. To remedy the shortcomings of these previous studies, we use a simple differential equations model without delays to examine temporal and frequency BR response to HUT for POTS patients. We use simulations to encode the three POTS phenotypes identified by Mar & Raj [3] to study how they affect BP and HR dynamics. Our model is formulated using a simple closed-loop zero-dimensional (0D) cardiovascular model, with first-order set-point control equations representing the BR regulation. We analyse our model output using signal processing techniques and study the effects of critical model parameters that correspond to the physiological abnormalities that cause each POTS phenotype. Results indicate that changes in clinically relevant parameters can generate low-frequency oscillations with amplitude equal to that observed in POTS patient data from our previous study [7]. Discussion of our results focuses on clinical implications and motivation for future studies.

## 2. Methods

This study develops a closed-loop 0D model describing the emergence and amplification of low-frequency (∼0.1 Hz) oscillations observed in POTS patients during HUT. The model is parameterized to fit average BP and HR signals measured in control and POTS patients, and simulation results are depicted along with characteristic data. Model results are predicted at rest and during HUT, and by varying characteristic parameters, we demonstrate how to differentiate POTS phenotypes suggested by Mar & Raj [3].

**Figure 1.**
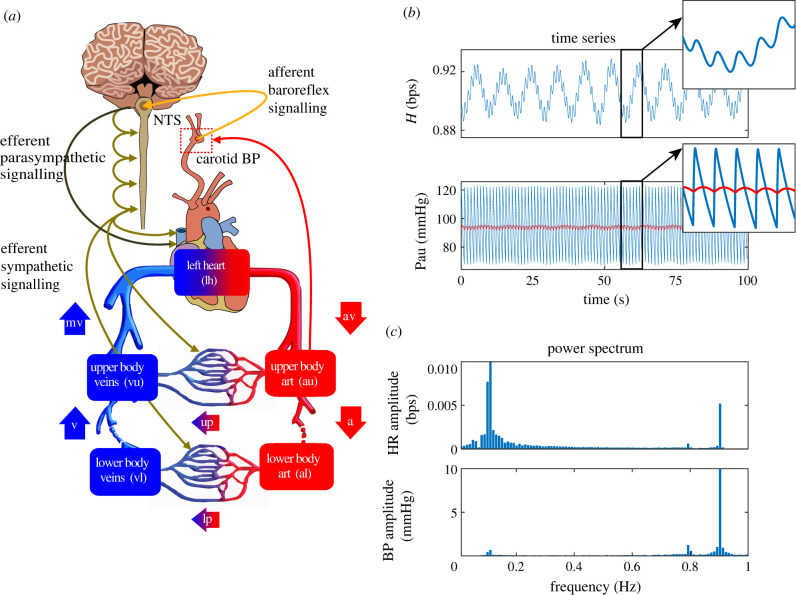
(*a*) Haemodynamics is controlled by the baroreflex system, which senses changes in carotid arterial pressure predicted as a function of upper body arterial pressure. Afferent signals from baroreceptor neurons are integrated into the nucleus solitary tract (NTS) and transmitted via sympathetic and parasympathetic neurons regulating HR, peripheral vascular resistance, and ventricular contraction. The systemic circulation is represented by compartments lumping upper (au) and lower (al) body arteries, upper (vu) and lower (vl) body veins and the left heart (lh). The upper body compartment contains organs above the lower abdomen, including the abdominal splanchnic vessels, and the lower body compartment contains organs below the lower abdomen. Flow (*Q*) through the aortic valve (av) is transported from the left heart to the upper body arteries. From here, it is transported to the lower body arteries and through the upper body peripheral vasculature (up) to the upper body veins. A parallel connection transports flow through the lower body peripheral vasculature (lp). From the lower body venous flow is transported to the upper body veins and finally via the mitral valve (mv) back to the left heart. Each compartment representing the heart or a collection of arteries or veins has a pressure (*P*), volume (*V*) and elastance (*E*). Pumping of the heart is achieved by assuming that left heart elastance (*E*_lh_(*t*)) is time-varying. (*b*) Model predictions of heart rate (*H* (bps), top panel) and upper body arterial pressure (*P*_au_ (mmHg), lower panel). We analyse upper body arterial pressure for oscillations but use the mean carotid pressure (not shown) as the input to our control equations. Five second sections of each signal are shown in the overlaid subpanels. (*c*) Frequency spectra of time-series data (*H* top, *P*_au_ bottom) shown in (*b*).

Similar to our previous studies [21,28], we predict blood flow and pressure in the systemic circulation using an electrical circuit model with five compartments, including the upper and lower body arteries and veins, and the left heart (figure 1*a*). The BR is incorporated via negative feedback control equations predicting the effector response (HR, vascular resistance, and cardiac contractility) as functions of mean carotid pressure. The magnitude and phase of the low-frequency oscillations generated by the BR are determined using discrete Fourier transform, analysing computed HR and BP signals.

Computations are first conducted in the supine position, followed by HUT simulated by accounting for gravity shifting blood from the upper to the lower body. We demonstrate the importance of incorporating HR variability by adding uniformly distributed white noise to predictions of HR and discuss how phenotypes suggested by Mar & Raj [3] can be simulated. Computer code for the model simulations is available at https://github.com/msolufse/BaroreflexPOTSmodel.

### 2.1. Data

In this study, model simulations are qualitative and meant to illustrate how changing system properties impact dynamics. To test if the outcome of our simulations is realistic in terms of physiological behaviour, we included BP and HR measurements (extracted from [7]) from two representative subjects: a control subject and a POTS patient.

Measurements from these subjects include continuous electrocardiogram (ECG) and upper body arterial BP measurements extracted at rest for 5 min and then for an additional 5 min after the HUT onset. HR is extracted from the high-resolution three-lead ECG measurements as the inverse distance between consecutive RR intervals and continuous BP measurements are obtained using a Finapress device (Finapres Medical Systems BV, Amsterdam, Netherlands). BP and ECG signals are sampled at 1000 Hz. BP and HR signals are sub-sampled to 250 Hz, after which the uniform phase empirical mode decomposition [7] is used to extract the magnitude and phase of ∼0.1 Hz oscillations. Data are scaled to literature values in the supine position (at rest) for a healthy subject setting HR to 60 bpm = 1 bps and BP varying between 80 and 120 mmHg. In addition, to illustrate severe tachycardia characteristic of hyperadrenergic POTS, during HUT, HR is set and maintained at 1.5 bps, an increase of 30 bpm = 0.5 bps.

The afferent input to the BR control model assumes that BP is measured at the level of the carotid baroreceptors (above the centre of gravity). Therefore, BP data, measured at the level of the heart, is adjusted by subtracting the effect of gravity as described in our previous study [21].

### 2.2. Cardiovascular model

We employ an electrical circuit analogy to predict blood flow (analogous to current), pressure (analogous to voltage) and volume (analogous to charge) in the systemic circulation represented by five compartments, including the upper (*u*) and lower (*l*) body arteries (*a*) and veins (*v*), and the left heart (lh). Each compartment is quantified by its volume (*V*(*t*) ml) and pressure (*P*(*t*) mmHg), while flow (*Q*(*t*) ml s^−1^) exists between compartments. The lower body contains organs below the lower abdomen while the upper body compartments contain organs above the lower abdomen including the abdominal-splanchnic vessels. Figure 1 depicts the model and table 1 lists the dependent cardiovascular variables.

**Table 1.** Dependent variables including volume (*V* ml), pressure (*P* mmHg), and flow (*Q* ml s^−1^) for the cardiovascular system and baroreflex control system. The latter includes peripheral vascular resistance (*R*_up_ and *R*_lp_ mmHg s ml^−1^), left ventricular elastance (*E*_lv_ mmHg ml^−1^) and heart rate (*H* bps).

state variables			
symbol	description	states	units
*R*	resistance	upper peripheral (up)	mmHg s ml^−1^
		lower peripheral (lp)	mmHg s ml^−1^
*E*	elastance	left heart (lh)	mmHg ml^−1^
		diastolic value of left ventricle elastance (ED)	mmHg ml^−1^
*P*	pressure	left heart (lh)	mmHg
		arteries, upper (au)	mmHg
		arteries, lower (al)	mmHg
		veins, upper (vu)	mmHg
		veins, lower (vl)	mmHg
		mean (m)	mmHg
*V*	volume	left heart (lh)	ml
		arteries, upper (au)	ml
		arteries, lower (al)	ml
		veins, upper (vu)	ml
		veins, lower (vl)	ml
*Q*	flow	atrial valve (av)	ml s^−1^
		arteries (*a*)	ml s^−1^
		upper peripheral (up)	ml s^−^^1^
		lower peripheral (lp)	ml s^−1^
		veins (*v*)	ml s^−1^
		mitral valve (mv)	ml s^−1^
*H*	heart rate	—	bps

To ensure flow conservation, for each compartment (i=lh,au,al,vl,vu), the change in volume is computed as the difference between flow into and out of the compartment,

2.1 $$\frac{dV_{i}}{dt}=Q_{\mathrm{in}}-Q_{\mathrm{out}},$$

where $Q_{\mathrm{in}}$ denotes the flow into, and $Q_{\mathrm{out}}$ denotes the flow out, of compartment i. Ohm’s Law relates flow to pressure and the resistance (R, ${\mathrm{mmHg}\;s\;\mathrm{ml}}^{-1}$) between compartments (i−1) and (i),

2.2 $$Q_{i}=\frac{P_{i-1}-P_{i}}{R_{i}}.$$

For each arterial compartment and upper venous compartment i, pressure and volume are related using the linear relation

2.3 $$P_{i}-P_{ui}=E_{i}(V_{i}-V_{ui}),$$

where $V_{ui}$ is the unstressed volume, $E_{i}$ the elastance (reciprocal of compliance, analogous to capacitance) and $P_{ui}=0$ the unstressed pressure.

Given that pressure changes significantly on the venous side, in particular in the lower venous compartment during HUT, as suggested by Hardy *et al.* [29], we employ a nonlinear relation between lower venous pressure and volume given by

2.4 $$P_{\mathrm{vl}}=\frac{1}{m_{\mathrm{vl}}}\log\left(\frac{V_{\mathrm{Mvl}}}{V_{\mathrm{Mvl}}-V_{\mathrm{vl}}}\right),$$

where $m_{\mathrm{vl}}$ is a parameter that relates nominal pressure, volume ($V_{\mathrm{vl}}$) and maximal volume ($V_{\mathrm{Mvl}}$) [30]. The pumping of the heart is achieved by introducing a time-varying elastance function of the form

2.5 $$E_{\mathrm{lh}}(t)=\left\{ \begin{array}{cc} \frac{E_{S}-E_{D}}{2}\left(1-\cos(\frac{\pi t}{T_{S}})\right)+E_{D}\; & 0\leq t\leq T_{S}\;\\ \frac{E_{S}-E_{D}}{2}\left(\cos(\frac{\pi (t-T_{S})}{T_{D}})+1\right)+E_{D}\; & T_{S}\leq t\leq T_{S}+T_{D}\;\\ E_{D}\; & T_{S}+T_{D}\leq t\leq T,\; \end{array}\right.$$

where $E_{S},E_{D},T_{S}$ and $T_{D}$ denote the end systolic and end diastolic elastance, and the time for end systole and diastole, respectively. This function is used to model cardiac contraction determined by $E_{M}-E_{m}$.

The timing parameters $T_{S}$ and $T_{D}$ are determined as functions of the length of the current cardiac cycle (T, the RR interval). By combining the prediction of the length of the QT interval from [31,32] and the ratio of cardiac mechanical contraction to relaxation from [33], we get

2.6 $$T_{S}=0.45\left(c_{1}+\frac{c_{2}}{T}\right)\;\mathrm{and}\;T_{D}=0.55\left(c_{1}+\frac{c_{2}}{T}\right),$$

where $c_{1}=0.52$ s and $c_{2}=-0.11\;s^{2}$ from [31].

Similar to arterial compartments, the left heart pressure ($P_{\mathrm{lh}}$) and volume ($V_{\mathrm{lh}}$) are related by

2.7 $$P_{\mathrm{lh}}-P_{\mathrm{lh},u}=E_{\mathrm{lh}}(t)(V_{\mathrm{lh}}-V_{\mathrm{un}}),$$

where $P_{\mathrm{lh},u}=0$ and $V_{\mathrm{lh},u}=10$ are the unstressed pressure and volume in the left heart, and $E_{\mathrm{lh}}(t)$ is the time-varying elastance.

### 2.3. Head-up tilt

**Figure 2.**
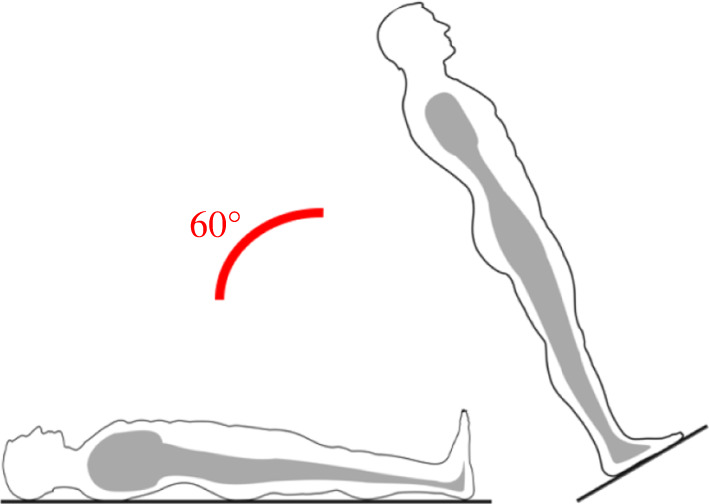
Head-up tilt (HUT) test. Patients are tilted, head up, from 0 to $60^{\circ }$ over 7 s. The shaded regions illustrate the blood volume distribution.

During HUT, gravity pools blood from the upper to the lower body affecting the flow between these compartments ($Q_{a}$ and $Q_{v}$). This manoeuvre is depicted in figure 2. We model this effect by adding a tilt term accounting for the additional force caused by gravitational pooling [21], i.e.

2.8 $$Q_{a}=\frac{P_{\mathrm{au}}-P_{\mathrm{al}}+P_{\mathrm{tilt}}}{R_{a}}\;\mathrm{and}\;Q_{v}=\frac{P_{\mathrm{vu}}-P_{\mathrm{vl}}-P_{\mathrm{tilt}}}{R_{v}},$$

with

2.9 $$P_{\mathrm{tilt}}=\rho gh\sin\left(\frac{\theta \pi }{180}\right),\;\theta \in [0^{\circ },\ldots ,60^{\circ }],$$

where $\rho =1.06\;[{g\;\mathrm{cm}}^{-3}]$ is the density of blood, $g=982\;[\mathrm{cm}\;s^{-2}]$ is the gravitational constant, h=25 [cm] is the height between the upper and lower body compartments, and θ is the angle of tilt. We determine the value of h by estimating the distance between the centre of mass for the upper body (the lower chest) and lower body (the pelvis) compartments.

### 2.4. Baroreflex model

The BR control system maintains homeostasis. Afferent baroreceptor nerves sense changes in the aortic arch and carotid sinus BP. Signalling in afferent baroreceptor neurons stimulated by BP are integrated into the medulla, from which efferent neurons are activated, modulating signalling along sympathetic and parasympathetic neurons. We do not compute sympathetic and parasympathetic outflow directly but instead predict controlled quantities as a function of pressure, i.e. afferent and efferent signalling lumps contributions from afferent and efferent (sympathetic and parasympathetic) nerves. Quantities controlled include HR (H), peripheral vascular resistance ($R_{\mathrm{up}},R_{\mathrm{lp}}$) and cardiac contractility ($E_{D}$).

As discussed in previous studies [13,21], during HUT, afferent BR firing is modulated by changes in mean carotid pressure. The cardiovascular model described above, predicts upper body arterial pressure at the level of the heart. Using analysis from Williams *et al.* [21], we compute carotid pressure from upper body arterial pressure as

$$P_{c}=P_{\mathrm{au}}-\rho g\tilde{h}\sin(\theta )$$

and mean carotid pressure as

2.10 $$\frac{d{\bar{P}}_{c}}{dt}=\frac{-{\bar{P}}_{c}+P_{c}}{\tau_{P}},$$

where $\tilde{h}=20\;[\mathrm{cm}]$ is the height between the carotid and aortic baroreceptors.

Equations for the BR control regulating effectors $X\in \{R_{up},R_{lp},E_{D},H\}$ (listed with units in table 2) as functions of mean carotid pressure (${\bar{P}}_{c}$) are derived under the assumption that each response has a saturation point and a minimum value. This assumption motivates the use of first-order kinetic control equations given by

2.11 $$\frac{dX}{dt}=\frac{-X+\tilde{X}({\bar{P}}_{c})}{\tau_{X}},$$

where $\tau_{X}$ is the time constant for the response X (shorter for effectors primarily modulated by the parasympathetic neurons than those primarily modulated by sympathetic neurons). For the control of HR (H) and vascular resistance ($R_{\mathrm{up}},R_{\mathrm{lp}}$) the set-point function $\tilde{X}$ is represented by a decreasing Hill function of the form

2.12 $$\tilde{X}=(X_{M}-X_{m})\frac{{\bar{P}}_{c}^{k_{X}}}{{\bar{P}}_{c}^{k_{X}}+P_{2X}^{k_{X}}}+X_{m},$$

while ventricular contractility is controlled by changing the minimum end diastolic elastance ($E_{D}$). For this control, the set-point function $\tilde{X}$ is represented by a decreasing Hill function of the form

**Figure 3.**
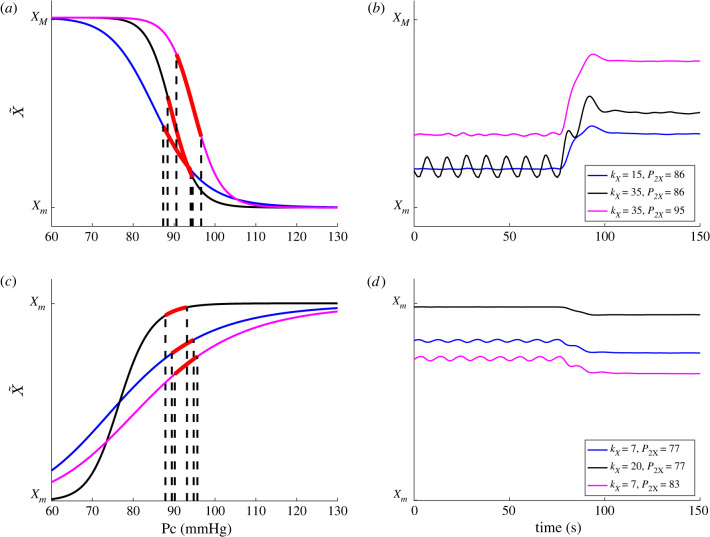
Left panels show the decreasing (*a*) and increasing (*c*) Hill functions (light blue, grey, light pink) for characteristic values of $k_{X}$ and $P_{2X}$. Model predictions using these functions are superimposed in (blue, black, pink). (*b*,*d*) The time series predicted from the differential equation (2.11) using the Hill functions in (*a*,*c*).

2.13 $$\tilde{X}=(X_{M}-X_{m})\frac{P_{2X}^{k_{X}}}{{\bar{P}}_{c}^{k_{X}}+P_{2X}^{k_{X}}}+X_{m},$$

where $X_{M}$ is the maximum value of $\tilde{X}$, $X_{m}$ is the minimum value, $P_{2X}$ is the half-saturation value and $k_{X}$ is the Hill coefficient. Graphs depicting the increasing and decreasing Hill functions, varying the steepness $k_{X}$ and the half-saturation value ($P_{2X}$), and how these impact predictions of effectors are shown in figure 3.

**Table 2.** Quantities controlled by the baroreflex system. Columns denote the quantity being controlled, the symbol characterizing the control, if the associated Hill function is increasing or decreasing, and units.

quantity being controlled	symbol	increasing/decreasing	units
resistance, upper peripheral	${\tilde{R}}_{\mathrm{up}}$	decreasing	$\mathrm{mmHg}\;s\;{\mathrm{ml}}^{-1}$
resistance, lower peripheral	${\tilde{R}}_{\mathrm{lp}}$	decreasing	$\mathrm{mmHg}\;s\;{\mathrm{ml}}^{-1}$
elastance at end diastole	${\tilde{E}}_{D}$	increasing	$\mathrm{mmHg}\;{\mathrm{ml}}^{-1}$
heart rate	$\tilde{H}$	decreasing	bps

It should be noted (as shown in figure 3) that the controls are not initiated at half the maximum value but at values ensuring that the controlled effectors can increase and decrease as expected from physiological considerations. Additionally, we see that while the response curve, $\tilde{X}$, may take on these saturated values, the actual response does not. This can be seen in figure 3 where pressure changes from 85 to 100 mmHg which causes $\tilde{X}$ to vary between the lower ($X_{m}$) and upper ($X_{M}$) bounds. Figure 3 shows this for varying values of $k_{X}$ and $P_{2X}$.

The equations listed above provide continuous estimates for the effector variables, which modulate the equations relating the dependent cardiovascular variables. The peripheral resistances ($R_{\mathrm{up}},R_{\mathrm{lp}}$) are used in equation (2.1) relating flow to pressure. End diastolic elastance ($E_{D}$) is used in equation (2.5) predicting the heart’s pumping, and HR (H) is used to determine the length of the cardiac cycle ($T=1/H_{b}$).

Note that T is a discrete quantity only updated at the end of each heart beat, while H is a continuous variable. To ensure that T remains discrete, we introduce a discrete HR ($H_{b}$) that remains constant between cycles. For the first cardiac cycle (i=1, at time t=0) $H_{b}^{1}=H(0)$ and $T_{i}=1/H_{b}^{1}$. For subsequent cardiac cycles i>1, $H_{b}^{i}=H(t_{\mathrm{end}}^{i-1})$, where $t_{\mathrm{end}}^{i-1}$ is the time at the end of the previous cycle and $T^{i}=1/H_{b}^{i}$.

### 2.5. Heart rate variability

In addition to changes in BP mediated by the BR control system, HR data exhibit spontaneous variation, referred to as heart rate variability (HRV) [34] likely caused by fluctuations in vagal firing. These oscillations have physiological relevance and have proven to be essential for cardiovascular dynamics. Typically healthy young people have a high HRV, while the elderly and people with autonomic dysfunction have low HRV. However, setting up a mechanistic model predicting HRV is challenging. Most studies accounting for HRV refer to the phenomena as mathematical chaos [35,36], and while it is believed to most closely resemble ‘pink noise’ [37], as suggested by others, we use ‘white noise’ to predict HRV.

As noted above, we solve the differential equation (2.12) continuously and update $H_{b}$ and the length of each cardiac cycle ($T=1/H_{b}$) at the end of each cycle. HRV is accounted for by adding white noise to T sampled from a uniform distribution, i.e. we let

2.14 $$T{|}_{tH0}\leftarrow \frac{1}{H_{b}{|}_{t_{H0}}}\left(1+\frac{U[-1,1]}{50}\right),$$

where U[−1,1] is a uniform random distribution from −1 to 1, and $t_{H0}$ denotes the starting time for each heartbeat. We choose to scale the noise by 2% to approximately match patient data.

### 2.6. Model parameters and initial conditions

Nominal parameter values and initial conditions are deduced from literature and physiological data representing a healthy young female. Below we describe *a priori* calculation of the parameters, which are detailed with units in the tables 5 and 6.

#### 2.6.1. Cardiovascular parameters

**Blood volume** is calculated using height and body mass index (BMI). Combining the classic formula for BMI [38] by which weight is given by $W=\mathrm{BMI}{\left(h/100\right)}^{2}$, with Nadler’s equation for blood volume (BV) [39], to estimate BV (ml) as

2.15 $$\text{BV}=\left\{ \begin{array}{cc} 0.4948\;{\text{BMI}}^{0.425}\;h^{1.575}-1954, & \text{female},\\ 0.4709\;{\text{BMI}}^{0.425}\;h^{1.575}-1229, & \text{male}, \end{array}\right.$$

where h is the height in centimetres. For our baseline patient, we use the average height of women in Denmark, 167.2 cm [40], and a ‘healthy’ BMI of $22\;\mathrm{kg}\;m^{-2}$ [38].

The total BV is distributed between the systemic (85%) and pulmonary (15%) circulations [13]. Within the systemic circulation, at rest, we assume that 15% is in the arteries and 85% is in the veins. We assume that half the adipose tissue, one-fourth of the gastrointestinal tract, half of the muscle, half the skin and half of the skeleton are in the lower body while the rest of the organs (heart, half of muscle, brain, etc.) are located in the upper body. In the supine position, we assume that 80% of the blood is in the upper body, [13]. To predict circulating BV, we differentiate the volume between stressed (circulating) and unstressed volume. Following Beneken & DeWitt [41], in the arteries, we assume that 30% of the volume is stressed, while in the veins, we assume that 7.5% of the total volume is stressed.

**Blood pressure:** The model is parameterized to represent dynamics in a healthy young female with a systolic arterial pressure of 120 mmHg and diastolic arterial pressure of 80 mmHg [42]. Using standard clinical index [43], we compute the mean pressure as $P_{m}=(2/3)P_{\mathrm{dia}}+(1/3)P_{\mathrm{sys}}\approx 93$ mmHg [44]. As BP is typically measured in the arm, which is included in the upper body arteries, we assign these values to the upper arterial compartment. To allow blood flow from the upper to the lower body arteries, we set the lower body artery pressure to 0.98 times the values in the upper body. Since the venous circulation’s pulse pressure is small, we only determine mean values in venous compartments. Using standard literature values [13] we assume that the upper body venous pressure is 3 mmHg; again, to ensure flow in the correct direction, the lower body venous pressure is $P_{\mathrm{vl}}=1.1P_{\mathrm{vu}}$.

Parameters for the lower body venous pressure-volume equation (2.4) are calculated as

$$V_{\mathrm{Mvl}}=4V_{\mathrm{vlI}}$$

and

$$m_{\mathrm{vl}}=\frac{1}{P_{\mathrm{vlI}}}\log\left(\frac{V_{\mathrm{Mvl}}}{V_{\mathrm{Mvl}}-V_{\mathrm{vlI}}}\right),$$

where $V_{\mathrm{vlI}}$, $P_{\mathrm{vlI}}$ and $V_{\mathrm{Mvl}}$ is the nominal volume, pressure and maximal volume for the lower venous compartment (vl), respectively. $V_{\mathrm{Mvl}}$ is set such that the volume does not saturate at HUT, and $m_{\mathrm{vl}}$ is set such that at rest, blood flows from the lower to the upper body veins.

**Elastance:** To calculate the nominal elastance parameters for the arterial compartments and the upper body veins, we use equation (2.3), assuming that the unstressed pressure ($P_{\mathrm{un}}=0$) and using the stressed volume fractions given above. The nonlinear venous pressure–volume equation (2.4) was used to predict the lower venous elastance. This parameter is adjusted following the HUT onset to capture the effect of the changing pulse pressure during HUT.

*Left heart end-diastolic and end-systolic elastance:* At the end of diastole, the left heart pressure is approximately equal to the venous pressure, and the ventricular volume is maximal, i.e. the nominal (minimal) elastance at diastole can be approximated by $E_{D}=P_{\mathrm{vu}}/\max(V_{\mathrm{lh}})$. Similarly, at the end of systole, the left ventricular pressure is approximately equal to the arterial pressure, and the volume is minimal. Hence, the nominal (maximal) elastance at systole is given by $E_{S}=P_{\mathrm{au}}/\min(V_{\mathrm{lh}})$.

**Blood flow:** In a healthy human cardiac output (CO) is ∼$5\;l\;\min^{-1}$ [13]. We assume that the total BV is circulated in ∼60 s, i.e. the CO $\approx \mathrm{BV}/60\;\mathrm{ml}\;s^{-1}$ [21]. With our previously assumed distribution of blood, we estimate that 80% of CO travels through the upper peripheral, perfusing the upper body, while 20% perfuse the lower body [45]. Hence we obtain nominal values of $Q_{\mathrm{up}}=0.8\;\mathrm{CO}$, and $Q_{a}=Q_{\mathrm{lp}}=Q_{v}=0.2\;\mathrm{CO}$.

**Resistance:** The atrial and mitral valve resistance are both set to 0.0001, as we assume the valves do not have significant resistance compared to resistance generated by flow through the vasculature. Therefore, the remaining nominal resistances are calculated using Ohm’s Law, $R=(P_{i-1}-P_{i})/Q$, where $P_{i-1}$ is the pressure in the previous compartment, $P_{i}$ is the pressure in the destination compartment and Q is the flow.

#### 2.6.2. Baroreflex control parameters

Each control equation has four parameters, $\tau_{X},\;X_{M},\;X_{m}$ and $P_{2X}$. The time constant, $\tau_{X}$, represents the ratio of the speed of the neurological responses and the physiological control. The fast HR control is achieved by stimulating the parasympathetic system modulating HR within a few beats, followed by input from the sympathetic system modulating HR on the timescale of 12.5 s. Cardiac contractility and peripheral vascular resistance are primarily modulated by the sympathetic system acting on a timescale of 12.5 s [13]. Our model lumps sympathetic and parasympathetic stimulation. Therefore, we assume that

$$\tau_{H}=6.25<\tau_{E}\approx \tau_{R}=12.5.$$

The maximum and minimum values for the Hill functions are set using literature ensuring that both are above/below actual observations as seen in patients with postural tachycardia syndrome. For HR values of 160–170 bpm may be encountered [4] and in POTS patients with reflex syncope [46] HRs below 40 bpm are not uncommon [47].

We allow peripheral vasculature to dilate to 1.5 times the resting radius and constrict to 0.75 times the resting radius. To relate these measurements to resistance, we recall Poseuille’s Law, which state that resistance changes in proportion to the fourth power of the radius. Thus we assume that $R_{m}=0.2R_{I}$ and $R_{M}=3R_{I}$. To estimate $E_{DM}$, we refer to the increased potassium levels, which increase cardiac contractility [48]. From this work, we estimate the extent to which contractility can increase under stress and assume that maximum end-diastolic elastance control ($E_{DM}$) can increase to 125% of the initial value. In principle, the heart can relax completely by a lack of stimulus. We, therefore, set the minimum end-diastolic elastance control ($E_{Dm}$) to 1% of the initial value.

Half-saturation values are calculated from initial conditions assuming that the subject is at rest, i.e. at ${\bar{P}}_{c}={\bar{P}}_{c}(0),(dX/dt)=0$. Using this assumption together with estimates for the maximum and minimum response the half-saturation values $P_{2X}$ can be estimated from

$$P_{2X}={\bar{P}}_{c}(0)\left(\frac{X_{M}-X(0)}{X(0)-X_{m}}\right),\;X=E_{D},$$

and

$$P_{2X}={\bar{P}}_{c}(0)\left(\frac{X(0)-X_{m}}{X_{M}-X(0)}\right),\;X=R_{\mathrm{up}},R_{\mathrm{lp}},H.$$

#### 2.6.3. Initial conditions

The differential equations for the cardiovascular model in equations (2.1)–(2.5) tracking BV are initiated at end diastole, i.e. the ventricular volume is maximal. We assume that both control and POTS patients have a healthy heart with left ventricle volumes that are representative of this normal state (see table 5 for values). We also assume a physiologically healthy resting initial HR of 1 bps. The remaining initial conditions are set to the calculated nominal values.

### 2.7. Signal processing

**Figure 4.**
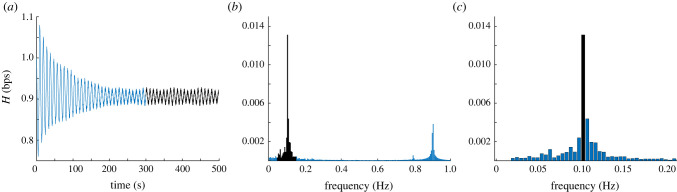
Process of obtaining amplitude of the ∼0.1 Hz component of a heart rate signal, the same process is used for blood pressure. Starting from the left, the solution obtained using a variable step size solver is interpolated at 100 Hz. The last 200 s are marked (black trace in (*a*)). Second, the discrete Fourier transform is applied to obtain the amplitude of the frequencies. We examine the frequency range corresponding to the baroreflex, 0.05–0.15 Hz (black spectrum in (*b*)). Third, we find the maximum of the amplitude in this range (black column in (*c*)).

We employ stationary signal processing to characterize oscillations seen in the model output. This process is illustrated in figure 4. We first solve the differential equations using MATLAB's [49] ode15s over a time interval long enough to ensure that all transient effects have died out. We interpolate over the solution to obtain a time series sampled uniformly at 100 Hz. We select the last 200 s of the H and $P_{\mathrm{au}}$ time series and compute the one-sided power spectrum using MATLAB's FFT algorithm. The design of our model suggests two explainable oscillations: one representing the BR, which operates at ∼0.1 Hz, and the HR, which operates at ~ 1 Hz. As shown in figure 4, these two oscillations and their harmonics are the only significant spikes in the frequency domain. To quantify the magnitude of oscillations caused by our control equations, we record the power and phase of the maximum amplitude peak in the ∼0.1 Hz frequency range. This process is applied at rest and during HUT.

### 2.8. Emergence of low-frequency oscillations

To capture the emergence of low-frequency ∼0.1 Hz oscillations, we first conduct a parameter sweep changing all relevant parameters over their physiological range. Specific emphasis is on parameters in equations (2.11–2.13) facilitating the BR control. This analysis is done in two steps, first detecting what parameters impact the dynamic behaviour and second conducting a detailed analysis varying the critical parameters that impact the dynamic response. In addition to detecting what parameters cause the model to change behaviour, we also investigate how to set parameters to capture oscillations at the ∼0.1 Hz frequency range.

Pseudo-code for this analysis is included in Algorithm 1. After solving the model for 250 s, the solution is examined to verify that steady state has been achieved for 200 s. To verify this, the interval is split in half, and the maximum and minimum HR (H) and upper arterial BP ($P_{\mathrm{au}}$) are calculated for each half. The relative difference between values for each half is computed and compared to a threshold (α). The halves are then interpolated at 100 Hz, and Fourier power spectra are computed for HR and BP for each half. The maximum ∼0.1 Hz power value is recorded for each half, and the relative difference between the power of the halves is computed and compared to a threshold (β). If all of these relative differences are less than their respective thresholds, the model is said to be in steady state, and the power spectra of the last 200 s are computed and recorded. If at least one of the relative differences is above the threshold, the model is solved for 20 additional seconds and checked for steady state behaviour again.

**Algorithm 1.** Pseudo code for two-dimensional parameter analysis. $H([T_{i},T_{k}])$ represents the value of heart rate (H) between time of $T_{i}$ and $T_{k}$, similarly for blood pressure ($P_{\mathrm{au}}$). $A_{H1}$ denotes the amplitude of the ∼0.1 Hz component of the H signal during $\left[T_{0},T_{1}\right]$ as is explained in methods §2.7. Similarly, $A_{P1}$ for $P_{\mathrm{au}}$. $A_{H2}$ and $A_{P2}$ are denote the amplitude of the ∼0.1 Hz component during $\left[T_{1},T_{2}\right]$. Analysis is conducted for both rest and head-up tilt sections.

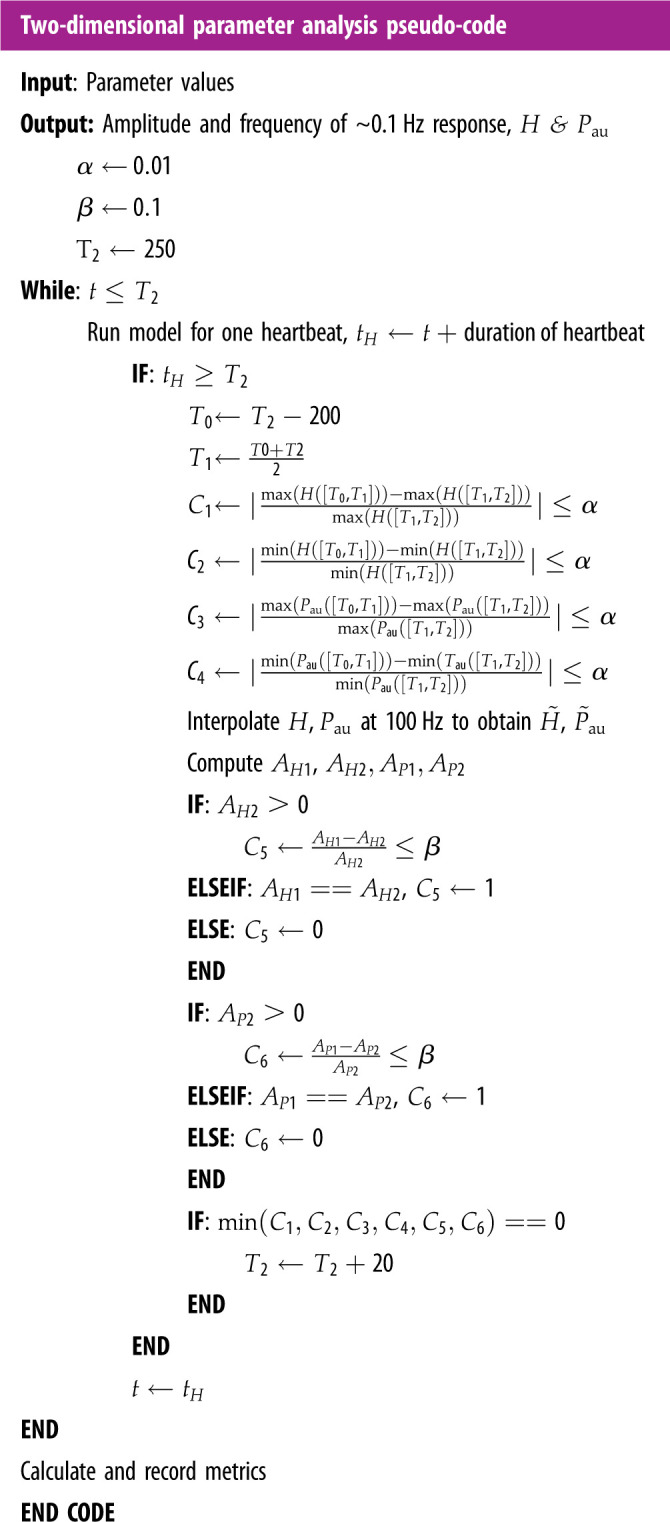

Using this automated parameter exploration approach, we map Hopf bifurcations for the low-frequency oscillations and the magnitude of HR and BP oscillations.

### 2.9. POTS phenotypes

We model the hyperadrenergic, neuropathic and hypovolaemic phenotypes of POTS suggested by Mar & Raj [3] by adjusting parameters (listed in table 3) reflecting hypothesized pathophysiology.

**Table 3.** Values of selected parameters before and after head-up tilt for phenotype simulations. Parameters are as follows: $C_{\mathrm{au}}$—upper arterial compliance, $P_{2H}$—half-saturation value for heart rate control, $k_{H}$—Hill coefficient for heart rate control, $k_{R}$—Hill coefficient for resistance control, $k_{E}$—Hill coefficient for left heart end diastolic elastance control, $R_{\mathrm{lpM}}$—maximum value of resistance control, $R_{\mathrm{lpm}}$—minimum value of resistance control.

phenotype	parameter	value before tilt	value after tilt
control	$C_{\mathrm{au}}$	$1.7\;\mathrm{ml}\;{\mathrm{mmHg}}^{-1}$	$0.86\;\mathrm{ml}\;{\mathrm{mmHg}}^{-1}$
	$P_{2H}$	88 mmHg	87 mmHg
hyperadrenergic	$k_{H}$	24 N.D.	34 N.D.
	$k_{R}$	23 N.D.	32 N.D.
	$k_{E}$	7 N.D.	10 N.D.
	$P_{2H}$	88.5 mmHg	89.8 mmHg
	$C_{\mathrm{au}}$	$1.7\;\mathrm{ml}\;{\mathrm{mmHg}}^{-1}$	$0.86\;\mathrm{ml}\;{\mathrm{mmHg}}^{-1}$
neuropathic	$R_{\mathrm{lpM}}$	$4.5\;\mathrm{mmHg}\;s\;{\mathrm{ml}}^{-1}$	$2.9\;\mathrm{mmHg}\;s\;{\mathrm{ml}}^{-1}$
	$R_{\mathrm{lpm}}$	$0.30\;\mathrm{mmHg}\;s\;{\mathrm{ml}}^{-1}$	$0.19\;\mathrm{mmHg}\;s\;{\mathrm{ml}}^{-1}$
	$C_{\mathrm{au}}$	$1.72\;\mathrm{ml}\;{\mathrm{mmHg}}^{-1}$	$0.86\;\mathrm{ml}\;{\mathrm{mmHg}}^{-1}$

**Hyperadrenergic POTS** is characterized by high levels of circulating norepinephrine during postural change, allowing the sympathetic nervous system to respond more to BP changes. To simulate this, at the HUT onset, we further increase parameters associated with sympathetic response, including $P_{2H}$ and $k_{H},k_{E},k_{R}$.

**Neuropathic POTS** is caused by partial neuropathy of the lower body vasculature, which causes abnormal blood pooling in the lower extremities. To simulate this, we decrease the control for the lower body resistance by reducing $R_{\mathrm{lpM}}$ and $R_{\mathrm{lpm}}$ after HUT.

**Hypovolaemic POTS** is obtained by decreasing the total BV. In our model, BV is calculated as a function of BMI (equation (2.15)). Simulations are conducted by changing BMI from 28 (large BV 4500 ml for an overweight young female) to 19 (representing an underweight young female, BV 3500 ml). The latter corresponds to the hypovolaemic patient group discussed by Mar & Raj [3]. A low BV alone does not compromise the BR and therefore only represents a POTS phenotype if the patient is experiencing POTS symptoms. Many POTS patients with severe symptoms are young skinny female patients. Therefore, in addition to investigating the isolated effect of low BV, we study how changes in BV affect hyperadrenergic and neuropathic POTS patients.

To allow for a smooth parameter transition during HUT, we include a 10-s delayed onset, i.e. we let

2.16 $$x=\left\{ \begin{array}{cc} x_{0}\; & t<t_{\mathrm{HUT}}+10,\;\\ (x_{1}-x_{0})\frac{{\left(t-t_{\mathrm{HUT}}-10\right)}^{8}}{{\left(t-t_{\mathrm{HUT}}-10\right)}^{8}+5^{8}}+x_{0}\; & t\geq t_{\mathrm{HUT}}+10,\; \end{array}\right.$$

where x is the parameter being changed after HUT, $x_{0}$ is the value during rest, $x_{1}$ is the value of the parameter that is being transitioned to and $t_{\mathrm{HUT}}$ is the time of the HUT onset.

## 3. Results

Results demonstrate the emergence of low-frequency oscillations at rest and during HUT and how the phenotypes proposed by Mar & Raj [3] can be simulated.

### 3.1. Low-frequency oscillations

Our model, shown in figure 1, can generate ∼0.1 Hz HR (H) and BP ($P_{\mathrm{au}}$) oscillations observed in patient data [7]. The amplitude and frequency of the oscillations can be modulated by varying the model parameters in the BR control equations (2.11–2.13), including the maximum $X_{M}$ and minimum $X_{m}$ response, the time constants $\tau_{X}$, the half-saturation values $P_{2X}$ and the Hill-coefficients $k_{X}$, X=H, R, E.

**Oscillation frequency** is primarily determined by time constants ($\tau_{X}$) differentiating the parasympathetic and sympathetic control. Efferent responses mediated by the parasympathetic system are significantly faster than those transmitted via the sympathetic system [13], i.e. $\tau_{H}\ll \tau_{R}=\tau_{E}$. The ∼0.1 Hz frequency was achieved using time constants reported in table 6. The Hill coefficients ($k_{X}$) also affect frequency but to a lesser extent than $\tau_{X}$.

**Oscillation amplitude** can be modulated by changing $k_{X},P_{2X}$ and $X_{M}-X_{m}$, X=H, R, E. We studied the effect of varying all parameters over their physiological range. Results (summarized in table 4) show that $k_{X},\;X=H,\;R,\;E$ impact the oscillation amplitude, with $k_{H}$ being the most influential parameter. Increasing the Hill coefficients $k_{X}$ increases the sensitivity of the BR control. A larger value of $k_{X}$ gives a steeper Hill function (shown in figure 3), i.e. the change in pressure needed to generate a given response decreases. Shifting the Hill function by changing the half-saturation value ($P_{2X}$) causes the operating regime to change to a steeper portion of the Hill function. This shift has the same effect as increasing $k_{X}$ but has a much smaller effect on the oscillation amplitude.

**Table 4.** Effects on heart rate (H) and upper arterial blood pressure ($P_{\mathrm{au}}$) when increasing stated parameter.

increased parameter	effect
$k_{H}$	increases H & $P_{\mathrm{au}}$ oscillation amplitude
$k_{R}$	increases H & $P_{\mathrm{au}}$ oscillation amplitude
$k_{E}$	increases H & $P_{\mathrm{au}}$ oscillation amplitude
$P_{2H}$	increases H, increases diastolic $P_{\mathrm{au}}$
$P_{2R}$	decreases H, increases $P_{\mathrm{au}}$
$P_{2E}$	decreases H, increases H & $P_{\mathrm{au}}$ oscillation amplitude
	increases $P_{\mathrm{au}}$ pulse-pressure
$H_{M}-H_{m}$	increases H & $P_{\mathrm{au}}$ oscillation amplitude
$R_{M}-R_{m}$	increases H & $P_{\mathrm{au}}$ oscillation amplitude
$E_{DM}-E_{Dm}$	increases H & $P_{\mathrm{au}}$ oscillation amplitude

**Figure 5.**
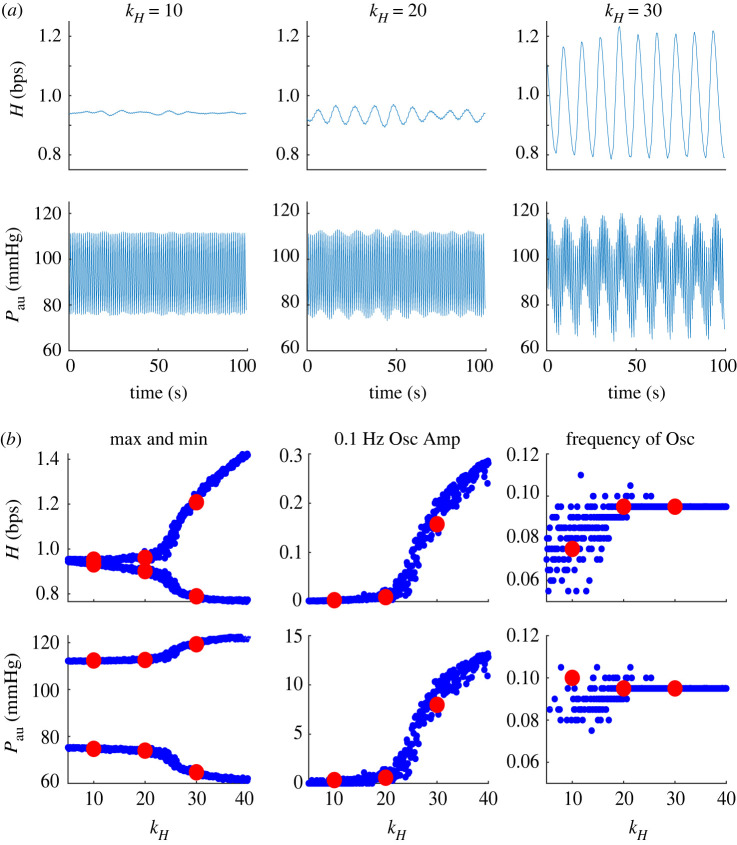
(*a*) From left to right: heart rate (H, top) and upper arterial pressure ($P_{\mathrm{au}}$, bottom) predictions for $k_{H}=10,\;20$ and 30. Note the large amplitude oscillations in the right panel are higher than values observed in patient data but are included to illustrate the behaviour of the model. (*b*) From left to right: maximum and minimum values for varying values of $k_{H}$, the amplitude of the ∼0.1 Hz region response, and frequency of oscillations. Enlarged red dotes show denote measurements corresponding to $k_{H}=10,20,30$.

Figure 5*a* (top panels) shows HR and BP dynamics in response to increasing $k_{H}$. We depict results of changing $k_{H}$, the most influential parameter, but similar results (not shown) are obtained when increasing $k_{R}$ and $k_{E}$, controlled by the sympathetic system. Results in figure 5*a* show small oscillations (left), medium oscillations that are approximately the size found in POTS patients (middle), and large oscillations, not likely to be observed in actual patients (right). For $k_{H}<6$, the system does not oscillate, at $k_{H}\approx 6$, oscillations emerge, and their amplitude increases with increasing values of $k_{H}$. Figure 5*b* depicts the change in amplitude and frequency as a function of $k_{H}$. Changing $k_{X},\;X=H,\;R,\;E$ impacts the oscillation amplitude more than frequency. The frequency almost doubles (from 0.06 to 0.11 Hz) while the HR oscillation amplitude increases from 0 to 0.3. The frequency diagrams in figure 5*b* (right column) have two characteristic features, a broad distribution (vertical spread) and horizontal stripes with white spacing. The former results from noise, added to HR, to account for HR variability and the latter from the frequency resolution. The model is solved with a time step of 0.01 s, with the Fourier transform calculated over a 200 s interval, giving a frequency resolution of 0.005 Hz.

**Figure 6.**
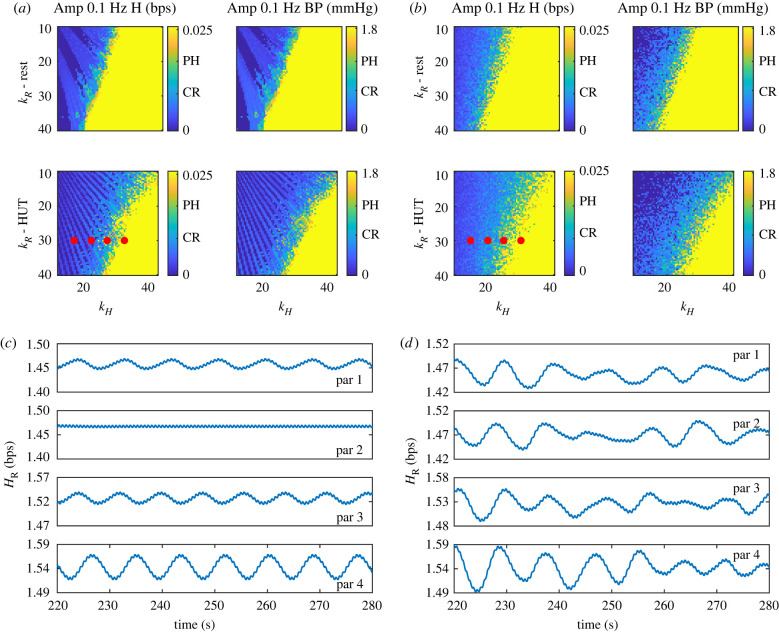
Two-dimensional parameter analysis of $k_{R}$ versus $k_{H}$. (*a*) Amplitudes of peak heart rate (H) oscillation (left) and peak upper arterial blood pressure ($P_{\mathrm{au}}$) oscillation (right) at the ∼0.1 Hz frequency band for values of $k_{R}$ and $k_{H}$ at rest (top) and head-up tilt (HUT, bottom). (*b*) The same information as (*a*) but with 2% noise. Average measurements from data [7] are marked for control patients at rest (CR), and POTS patients during head-up tilt (PH). Note that the physiologically possible oscillations correspond to the green regions. (*c*) Heart rate predictions during HUT for red dots on lower panels of (*a*); *i*th panel from top corresponds to *i*th dot from the left in (*a*). (*d*) Similar information as (*c*) but pertaining to (*b*).

As noted above, $k_{H}$ has the most significant impact on the system dynamics. Both the sympathetic and parasympathetic systems control HR, but as noted in the introduction, POTS may result from the expression of specific agonistic antibodies binding to $\beta_{1}$ and $\beta_{2}$ receptors [3]. Since these are found on pacemaker cells modulating HR and smooth muscle cells in the vasculature, we study the response to changing $k_{H}$ and $k_{R}$. Results shown in figures 6*a*,*b* reveal that increasing either $k_{H}$ or $k_{R}$ increases the amplitude of oscillations. This result agrees with the hypothesis that POTS patients have a more sensitive control system.

Other model parameters also change the dynamic behaviour—but not as significant as changes in $k_{X}$ (specifically $k_{H}$). In general, the half-saturation value offsets the control at different pressure levels but does not change the sensitivity, as the slope of the sigmoidal curve remains the same. Changing the range $\Delta X=X_{M}-X_{m}$ changes the width and steepness of the curve; the latter does have some effect on sensitivity, but it is not as significant as the effect observed when increasing $k_{X}$. Table 4 lists the impact of changing each parameter on HR and BP.

**Figure 7.**
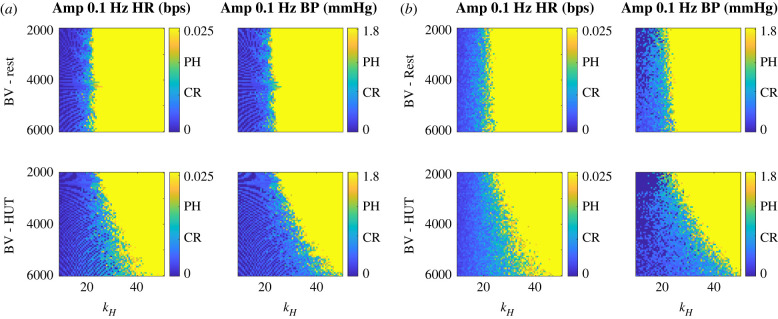
Two-dimensional parameter analysis of blood volume (BV) versus $k_{H}$. (*a*) Amplitudes of peak heart rate (H) oscillation (left) and peak upper arterial blood pressure ($P_{\mathrm{au}}$) oscillation (right) at the ∼0.1 Hz frequency band for values of BV and $k_{H}$ at rest (top) and head-up tilt (HUT, bottom). (*b*) The same information as (*a*) but with 2% noise. Average measurements from data [7] are marked for control patients at rest (CR), and POTS patients during head-up tilt (PH).

Lastly, figures 7*a*,*b* shows the effects of BV and $k_{H}$ on low-frequency oscillations. We see that, as before, larger values of $k_{H}$ result in larger oscillations. We also note that lower values of total BV contribute to larger low-frequency oscillations during rest and HUT.

### 3.2. Head-up tilt

**Figure 8.**
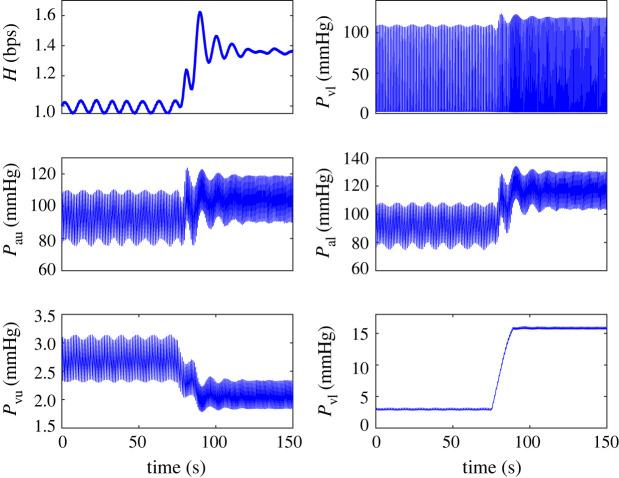
Results of simulation with HUT at t=75. Row 1: heart rate (H,  bps), left ventricle pressure ($P_{\mathrm{lv}}$, mmHg) row 2: upper arterial pressure ($P_{\mathrm{au}},$ mmHg), lower arterial pressure ($P_{\mathrm{al}},$ mmHg) row 3: upper venous pressure ($P_{\mathrm{vu}},$ mmHg), lower venous pressure ($P_{\mathrm{vl}},$ mmHg).

During HUT (shown in figure 2), gravity pools blood from the upper to the lower body, stimulating the autonomic nervous system. The result is a shift in BV and pressure, increasing in compartments below the centre of gravity and decreasing in compartments above. In our model, the upper body compartments are centred around the carotid baroreceptors, while the lower body compartments are centred in the lower part of the torso. Representative model BP predictions in all compartments are shown in figure 8. We note that after HUT, the pressure in the lower compartments increases while pressure in the upper compartments decreases. These simulations were generated with $k_{H}=27$, which causes the system to oscillate at rest and after HUT. Without changing parameters, oscillations dampen after HUT due to volume redistribution.

Like rest, control parameters impact predicted dynamics, and $k_{H}$ remains the most influential parameter. Figure 6*a* (bottom row) shows oscillation amplitude as a function of $k_{R}$ and $k_{H}$ without noise. For these simulations, the ‘non-oscillatory’ region appears striped, indicating bands of oscillations alternating with no oscillations. Figure 6*c* shows selected time-series predictions for parameter values marked on figure 6*a*. We note that in the non-oscillatory region, it is possible to increase $k_{H}$ and eliminate oscillations. These stripes are a result of the on–off behaviour of emerging low-frequency oscillations. Mathematically, this behaviour is common; as we change $k_{R}$ or $k_{H}$ the system undergoes repeated Hopf bifurcations. However, physiologically, small changes in a parameter have not been reported to affect the frequency response significantly. By adding noise mimicking HR variability (HRV) to the model, this behaviour disappears (the striped pattern disappears, see figure 6*b*), suggesting that the presence of HRV stabilizes the system response, as can be seen in figures 6*b*,*d*.

### 3.3. POTS phenotypes

Previous studies [1,3] suggest that POTS patients can be separated into neuropathic, hyperadrenergic, and hypovolaemic phenotypes. This section discusses how each of these can be represented in our model. The phenotype encoding is based on the assumption that the cardiovascular system of POTS patients changes in response to a postural change. For this reason, select parameters change after HUT to recreate dynamics. Depending on the phenotype, we select which parameters to change. We decrease the upper body arterial compliance for all simulations to account for volume redistribution upon HUT. The values of the changed parameters before and after HUT can be seen in table 3.

**Figure 9.**
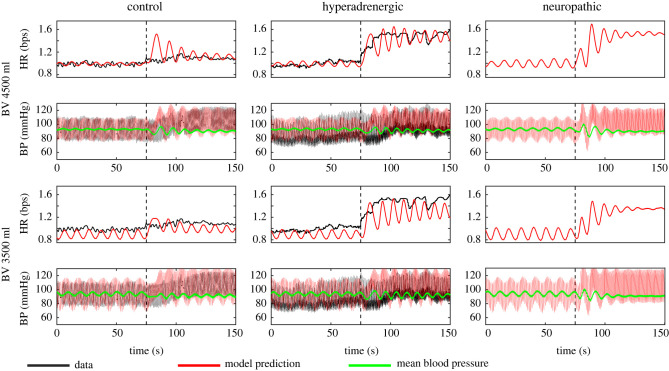
Characteristic data for a control and hyperadrenergic POTS patient (black) and model predictions (red and green) of heart rate (H), carotid artery blood pressure ($P_{c}$) and mean pressure. Simulations with 4500 ml of blood are in the top row with simulations with 3500 ml of blood in the bottom row. $C_{\mathrm{au}}$ is decreased after HUT for all simulations to represent constriction of vasculature upon HUT. In control $P_{2H}$ is decreased (left), $k_{H}$ and $P_{2H}$ are increased after HUT to replicate hyperadrenergic POTS (middle) and $k_{H}$ increased, $R_{\mathrm{lpM}}$ and $R_{\mathrm{lpm}}$ decreased to replicate neuropathic POTS (right). To compare model predictions H data are scaled such that the baseline is 1 bps and blood pressure vary from 80 to 120 mmHg.

**Control** subjects show a limited increase in HR and similar amplitudes of oscillations before and after HUT. When volume is redistributed during HUT, the BR control operating regime is shifted due to upper arterial pressure decreasing. To avoid an increase in HR, we shift the HR response curve with the pressure by reducing $P_{2H}$. Simulation of a control subject can be seen with data in the left column of figure 9.

**Hyperadrenergic POTS** patients have increased levels of plasma norepinephrine during HUT [3]. We model this by increasing $k_{i}$, i=H, E, R and $P_{2H}$ during HUT. Results, depicted in figure 9 (top row centre), show that increasing these parameters increases the amplitude of the ∼0.1 Hz oscillations (compared to the control subject—top left) and causes tachycardia during HUT, which is consistent with POTS patient data.

**Neuropathic POTS** patients experience excessive blood pooling below the thorax during HUT due to partial autonomic neuropathy. This condition is simulated by decreasing the range of resistance control in the lower body arteries, i.e. we reduce $R_{\mathrm{lpM}}$ and $R_{\mathrm{lpm}}$ making this control less effective. Results from this simulation depicted in figure 9 top right show that subjects exhibit tachycardia but that oscillations are dampened after HUT onset.

**Hypovolaemia** To understand how hypovolaemia impacts our predictions, we reduce central BV by lowering the BMI. We found that hypovolaemia alone cannot reproduce POTS dynamics—the model predicts low tachycardia values and no oscillations. This may be because our model cannot distinguish between healthy patients with low BV, who do not experience tachycardia, and POTS patients. Figure 9, bottom row, shows that HR is lower than in patients with a normal BV. However, for hyperadrenergic POTS patients, the HR and BP oscillations amplitude increase significantly, indicating that this patient group may experience a more severe response to POTS. To study this phenomenon further, we conducted a two-dimensional analysis examining the amplitude of HR and BP oscillations as a function of BV at rest and during HUT.

Figures 7*a*,*b* (top row) shows that reducing BV at rest does not impact dynamics. However, as can be seen in the bottom row of figures 7*a*,*b*, reducing BV during HUT increases oscillation amplitude. This implies that more severe oscillations occur during HUT for patients with less BV. Similar to figure 6, Hopf bifurcation lines can be seen in the parameter space in figure 7*a* but are removed when noise is added to simulations representing HR variability as can be seen in figure 7*b*.

## 4. Discussion

This study developed a closed-loop BR cardiovascular model and used simple signal processing to extract the frequency and amplitude of HR and BP oscillations. Results show that our model can generate oscillations in the low-frequency (∼0.1 Hz) range observed in control and POTS patients at rest and during HUT and that oscillations can be manipulated by modulating parameters associated with the BR.

Our model can predict tachycardia (an increase in HR of at least 30 bpm, 40 bpm in adolescents) observed in POTS patients by increasing the half-saturation of the HR response ($P_{2H}$) or decreasing the maximum and minimum vascular resistance ($R_{\mathrm{lpM}}$ and $R_{\mathrm{lpm}}$). The former is significantly more effective than the latter. Moreover, by changing physiologically relevant BR parameters after HUT, we can reproduce the hyperadrenergic and neuropathic POTS phenotypes suggested in [1,3]. Finally, we found that predictions are highly sensitive to changes in BV, suggesting that patients with low BMI, and low BV, may experience a more severe reaction than subjects with a healthy BMI and normal BV.

### 4.1. Low-frequency oscillations

The mathematical model used here extends previous studies [15,21,22,25,50–52] predicting cardiovascular dynamics using a closed-loop lumped parameter model including the left heart, the upper and lower body systemic arteries, and veins. The latter is included to facilitate the redistribution of volume upon postural change. The BR is modelled using a first-order control equation predicting the controlled quantity as a function of pressure using a sigmoidal function enforcing saturation at both high and low values of the controlled parameter.

By modulating parameters associated with the BR sensitivity (the sigmoidal $k_{X},\;X=R,\;E,\;H$), we explain the emergence and amplification of the low-frequency oscillations at rest and during HUT. Our findings agree with those reported in our previous study [7], noting that the low-frequency oscillations (sometimes referred to as Mayer waves) are observed in all subjects and that the oscillation amplitude is increased in POTS patients in particular following HUT. Our findings also agree with previous experimental studies that report more significant low-frequency oscillations in cerebral blood flow [8,9].

While this phenomenon has been discussed in studies using signal processing to examine HR and BP time series, only a few studies by Ottesen *et al.* [53], and Ishbulatov *et al.* [27] used closed-loop modelling to replicate this phenomenon. Both these studies explained the emergence of oscillations by introducing a delay in sympathetic response. By contrast, our model predicts the emergence of the ∼0.1 Hz oscillatory response without introducing delay differential equations. A key observation of this study is that oscillations can emerge for specific regions of the parameter space using only Hill functions for BR control and that oscillations do not rely on an explicit time delay. These findings agree with the hypothesis that POTS patients may have an abnormally sensitive BR control supporting the hypothesis that POTS is a central nervous system disorder. Specifically, we observed that $k_{H}$ is the most influential parameter for the oscillation amplitude. At $k_{H}<k_{critical}$, the system does not oscillate, but as $k_{H}$ increases, oscillations emerge via a Hopf bifurcation. In addition, we found that the BR time constants modulate the oscillation frequency.

To better understand how key physiological parameters modulate oscillation amplitude, we conducted a two-dimensional parameter analysis. Figure 6 shows that increased peripheral resistance and HR response sensitivities ($k_{R},k_{H}$) increase oscillation amplitude during rest and HUT. We see in figure 6 that increased BV decreases oscillation during HUT. This agrees with clinical insights from Klinik Mehlsen, Frederiksberg, Denmark that patients with smaller BV have more pronounced POTS symptoms.

### 4.2. Head-up tilt

HUT test is useful for diagnosing POTS [11]. For a patient tilted head up, gravity pools blood in the lower body. Since no active muscle contraction is invoked, this passive test clearly depicts the neural response to BV redistribution. Mathematically, we predict the tilt by accounting for the gravitational pooling of blood in the lower body as a function of the tilt angle.

A few modelling studies [21,25,27] have examined the response to HUT. Williams *et al.* [21] used an open-loop patient-specific model to predict arterial BP using HR as an input, while Heldt *et al.* [25] used a closed-loop cardiovascular model with set-point representations of the BR simulating HUT by increasing pressures in venous compartments, and Ishbulatov *et al.* [27] simulate HUT by increasing pressure to the lower body arteries and internal organs. The study by Williams *et al.* [21] did not examine low-frequency oscillations, and in the study by Heldt *et al.* [25] the low-frequency oscillations were dampened in less than 1 min after the onset of HUT, Ishbulatov *et al.* [27] successfully recreated low-frequency oscillations after HUT but only considered healthy subjects. While these studies were able to predict the HUT response, our model is the only one that can generate closed-loop stable oscillations that agrees with POTS patient data.

**Table 5.** Nominal patient values used to calculate parameter value and initial conditions.

symbol	description	value or equation	units	ref
h	height	167	cm	[40]
BMI	body mass index	19∗,28	${\mathrm{kg}\;m}^{-2}$	[38]
BV	total blood vol	$0.495{\mathrm{BMI}}^{0.425}h^{1.575}-1954$	ml	[39,61]
CO	cadiac output	BV/60	${\mathrm{ml}\;s}^{-1}$	[21]
H	heart rate	0.96	bps	
T	cardiac cycle	1/H	s	
volume distribution				
$V_{a}$	arteries	0.15 BV	ml	[13]
$V_{v}$	veins	0.85 BV	ml	[13]
$V_{\mathrm{ub}}$	UB supine	$0.8V_{i}^{**}$	ml	[45]
$V_{\mathrm{lb}}$	LB supine	$0.2V_{i}^{**}$	ml	[45]
$V_{M,\mathrm{lh}}$	max left heart	110	ml	[13]
$V_{m,\mathrm{lh}}$	min left heart	50	ml	[13]
unstressed volumes				
$V_{a,u}$	arteries	$0.7V_{a}$	ml	[41]
$V_{v,u}$	veins	$0.925V_{v}$	ml	[41]
$V_{\mathrm{lh},u}$	left heart	10	ml	[62]
nominal pressures				
$P_{\mathrm{au}}$	UB arteries	$\frac{2}{3}80+\frac{1}{3}120$	mmHg	[43]
$P_{\mathrm{al}}$	LB arteries	$0.99p_{\mathrm{au}}$	mmHg	[21]
$P_{\mathrm{vu}}$	UB veins	2.75	mmHg	[13]
$P_{\mathrm{vl}}$	LB veins	1.1 $p_{\mathrm{vu}}$	mmHg	[21]
$P_{\mathrm{lh}}$	left heart	2.5	mmHg	[13]

vol, volume; UB, upper body; LB, lower body.

*Hypovolumic patient.

^**^i = *a* arteries and i=v veins.

Specifically, we observe that low-frequency oscillations exist and persist during HUT. However, to get adequate pulse pressure and oscillation amplitude, it is necessary to decrease upper arterial compliance to account for the constriction of vasculature upon HUT. We hypothesize that this impact can be explained by pressure and volume redistribution. We allow select parameters to change after HUT to duplicate patient data depending on the POTS phenotype appropriately.

Our studies assume that the table is tilted up at a constant speed mimicking standard clinical protocols. However, as reported in several recent studies examining tipping points [54], and in the study by Kamiya *et al.* [55], the tilt speed may impact the emergence and amplitude of oscillations. This topic should be explored in detail in future modelling and experimental studies.

### 4.3. POTS phenotypes

POTS pathophysiology is complex and not completely understood. Several recent studies [1,3,11] speculate that POTS comprise multiple phenotypes including hyperadrenergic, neuropathic and hypovolaemic POTS. Several hypotheses describing each phenotype have been put forward without clearly denoting how these manifest changes in HR and BP time series. Hyperadrenergic POTS is believed to result from increased levels of circulating norepinephrine, while patients with neuropathic POTS have partial neuropathy in lower vascular beds. Finally, hypovolaemic POTS is simply described as POTS in patients with low BV. Additionally, autoantibodies against $\beta_{1},\;\beta_{2},\;\alpha_{1},\;M_{1},\;M_{2}$ receptors may be responsible for some cases of POTS [1,56].

In addition to analysing oscillations, we simulate the two main phenotypes and study how BP and HR change in patients with normal and low BV. To predict hyperadrenergic POTS, we increase $P_{2H}$ and $k_{X}$, X=H, E, R after HUT representing the increased plasma norepinephrine concentration during HUT. In the neuropathic case, we decrease the maximum and minimum response of the lower peripheral resistance ($R_{\mathrm{lpM}},\;R_{\mathrm{lpm}}$) to represent neuropathy in lower extremities [11]. We observe that increasing $P_{2H}$ in the hyperadrenergic case and reducing $R_{\mathrm{lpM}},\;R_{\mathrm{lpm}}$ in the neuropathic case are essential to the presence of orthostatic tachycardia while increasing $k_{X},\;X=H,\;E,\;R$ in the hyperadrenergic case is vital to the amplitude of low-frequency oscillations. Figure 9 shows minimal oscillations in the neuropathic phenotype. This motivates future work to examine whether all POTS phenotypes exhibit increased low-frequency oscillations in HR and BP or if large oscillations are unique to the hyperadrenergic phenotype.

**Table 6.** Nominal parameter values, their descriptions, value or equation, unit and references. Unit abbreviations are: millimetres of mercury (mmHg), seconds (s), millilitres (ml), beats per second (bps), non-dimensional (nd). The cardiovascular parameters were the same for all phenotypes simulated, while the baroreflex control parameters differ. Table 2 lists values used for each phenotype.

symbol	description	value or equation	units	ref
resistances				
$R{*}_{iv}$	valves	$1.0\times 10^{-4}$	${\mathrm{mmHg}\;s\;\mathrm{mmHg}}^{-1}$	
$R_{\mathrm{up}}$	UB periph	$\left(\;p_{\mathrm{lh}}-p_{\mathrm{au}}\right)/0.8\;\mathrm{CO}$	${\mathrm{mmHg}\;s\;\mathrm{mmHg}}^{-1}$	
$R_{a}$	UB→LB art	$\left(\;p_{\mathrm{lh}}-p_{\mathrm{au}}\right)/0.2\;\mathrm{CO}$	${\mathrm{mmHg}\;s\;\mathrm{mmHg}}^{-1}$	
$R_{lp}$	LB periph	$\left(\;p_{lh}-p_{au}\right)/0.2\;\mathrm{CO}$	${\mathrm{mmHg}\;s\;\mathrm{mmHg}}^{-1}$	
$R_{v}$	LB→UB veins	$\left(\;p_{\mathrm{lh}}-p_{\mathrm{au}}\right)/0.2\;\mathrm{CO}$	${\mathrm{mmHg}\;s\;\mathrm{mmHg}}^{-1}$	
compliances				
$C_{\mathrm{au}}$	art UB	$(V_{\mathrm{au}}-V_{\mathrm{au},u})/p_{\mathrm{au},\mathrm{dia}}$	${\mathrm{ml}\;\mathrm{mmHg}}^{-1}$	
$C_{\mathrm{al}}$	art LB	$(V_{\mathrm{al}}-V_{\mathrm{al},u})/p_{\mathrm{al}}$	${\mathrm{ml}\;\mathrm{mmHg}}^{-1}$	
$C_{\mathrm{vu}}$	veins UB	$(V_{\mathrm{vu}}-V_{\mathrm{vu},u})/p_{\mathrm{vu}}$	${\mathrm{ml}\;\mathrm{mmHg}}^{-1}$	
elastances				
$E_{ES}$	LH end systole	$p_{\mathrm{lh}},D/V_{M,\mathrm{lh}}$	${\mathrm{mmHg}\;\mathrm{ml}}^{-1}$	
$E_{ED}$	LH end diastole	$p_{au,S}/V_{m,lh}$	${\mathrm{mmHg}\;\mathrm{ml}}^{-1}$	
heart rate time constants				
$T_{S}$	end systole	0.45(0.52−0.11/T)	s	[31–33]
$T_{D}$	end diastole	0.55(0.52−0.11/T)	s	[31–33]
baroreflex sensitivities (Hill coefficients)				
$k_{R}$	resistance	23	N.D.	
$k_{E}$	LH end diastole	7	N.D.	
$k_{H}$	heart rate	27	N.D.	
baroreflex timescales				
$\tau_{R}$	resistance	12.5	s	
$\tau_{E}$	LH end diastole	12.5	s	
$\tau_{H}$	heart rate	6.25	s	
$\tau_{P}$	mean UB art BP	2.5	s	
baroreflex maximum (M) and minimum (m) saturations				
$H_{M}$	heart rate max	3.3	bps	[63]
$H_{m}$	heart rate min	0.3	bps	
$R_{lp,M}$	LB periph reist max	$3R_{lp}$	${\mathrm{mmHg}\;s\;\mathrm{mmHg}}^{-1}$	
$R_{\mathrm{lp},m}$	LB periph resist min	$0.2R_{\mathrm{lp}}$	${\mathrm{mmHg}\;s\;\mathrm{mmHg}}^{-1}$	
$R_{\mathrm{up},M}$	UB periph resist max	$3R_{\mathrm{up}}$	${\mathrm{mmHg}\;s\;\mathrm{mmHg}}^{-1}$	
$R_{\mathrm{up},m}$	UB periph resist min	$0.2R_{up}$	${\mathrm{mmHg}\;s\;\mathrm{mmHg}}^{-1}$	
$E_{D,M}$	LH ED elastance max	$1.25E_{D}$	${\mathrm{mmHg}\;s\;\mathrm{ml}}^{-1}$	[48]
$E_{D,m}$	LH ED elastance min	$0.01E_{D}$	${\mathrm{mmHg}\;s\;\mathrm{ml}}^{-1}$	
baroreflex half-saturation				
$P_{2H}$	heart rate	$p_{au}(H-H_{m})/(H_{M}-H)$	mmHg	
$P_{2R}$	periph resist	$p_{au}(R_{ip}-R_{ipm})/{\left(R_{ipM}-R_{ip}\right)}^{**}$	mmHg	
$P_{2E}$	LH elastance	$p_{au}(E_{DM}-E_{D})/(E_{D}-E_{Dm})$	mmHg	

UB, upper body; LB, lower body; LH, left heart; art, arteries;

periph, peripheral; LH, BP, blood pressure; ED, end diastole.

${\;}^{*}iv=av$ aortic valve, iv=mv mitral valve.

${\;}^{**}ip=up$ upper body peripheral, ip=lp lower body peripheral.

We could not reproduce the dynamics observed in POTS patient data by decreasing BV alone, likely because the model cannot distinguish POTS patients from healthy patients with a low BV. Figure 9 shows that low blood volume does not produce POTS dynamics from a control simulation but can make POTS dynamics more pronounced in simulations where the dynamics are already present. However, figure 7 shows that lower BV can result in larger oscillations during HUT. These findings imply that hypovolaemia may not be a distinct phenotype but exacerbates other phenotypes. More work is needed to study the effect of hypovolaemia, e.g. by introducing blood withdrawal or dehydration.

### 4.4. Heart rate variability

Several previous studies [35,36,57] have addressed the importance of HR variability. While there is still discussion on the origin of short-term HR variability [36], the net effect appears as noise. This study accounted for HR variability by adding noise to the predicted HR. The addition of HR variability stabilizes predictions eliminating frequent Hopf bifurcation lines seen in the top row of figures 6 and 7. The benefits of added noise in dynamic systems with stable fixed points have been shown in [58].

### 4.5. Importance of the study

This is the first study that uses a closed-loop model of the BR response to explain the emergence of low-frequency HR and BP oscillations observed in both control and POTS patients, along with the increase in amplitude observed in POTS patients during HUT. The presented model is the first attempt at representing the phenotypes of POTS using a mechanistic framework. Our results advance previous results [16,26,27] by describing oscillations during HUT with amplitudes consistent with POTS.

The clinical significance of this model is that it can encode the POTS phenotypes. Therefore, our study provides support for the current hypothesized mechanisms of POTS. We were able to show that hypovolaemia contributes to more severe oscillations, which could be linked to more severe symptoms when combined with the other phenotypes. However, we could not recreate POTS dynamics by decreasing BV alone. We also observed that the neuropathic phenotype resulted in tachycardia upon HUT but not increased oscillations. We successfully recreated observed dynamics by encoding the hyperadrenergic phenotypes into the model.

### 4.6. Limitations

Limitations of this work include the oversimplification of the vascular and BR model. The vascular model only includes the systemic circulation and separates the body into an upper and lower body compartment. This is not anatomically correct, as the body is a continuous cylinder; however, this assumption allows us to approximate blood flow in a simplified manner. The BR forms a complex negative feedback loop with numerous components, including the baroreceptors, afferent nerves, the NTS located in the medulla oblongata, efferent nerves, and the actual cell response in the sinoatrial node as well as muscle cells. Lumping these components into four control equations is a large assumption but is done to show that oscillations can be produced even with a simple model.

Another component not accounted for is respiration, which modulates BP directly by changing tissue pressure in the thorax [59] and indirectly via the respiratory sinus arrhythmia [60] modulating parasympathetic signal. The former adds an ∼0.25 Hz oscillation in BP and HR. The latter augments parasympathetic feedback adding a similar frequency component. As mentioned above, the model incorporates these features, but given the frequency separation, we do not anticipate they alter the principal results reported here addressing emergence and augmentation of ∼0.1 Hz oscillations. This model aims to provide a simple mathematical formulation to explore the possible origins of POTS. However, more intricate models accurately representing the actual physiology are needed for further exploration.

This study simulated HR variability by adding white noise to predictions of HR at a magnitude informed by data. Adding white noise allowed us to stabilize predictions, but more work is needed to investigate if HRV can be represented by white noise. As suggested by Goldberger *et al*. [37], it may be more appropriate to present HRV with pink noise.

Furthermore, we cannot predict the drop in arterial BP immediately after HUT as observed in the data, which is most likely due to the contraction of abdominal muscles and partly a result of the Valsalva manoeuvre as a reflex activity during positional changes. We also note that the data shown are exemplary and there was no attempt to estimate parameters based on these data. This oversimplification hinders the model in predicting the precise hypotheses of the origin of POTS, such as the exact type of hyperadrenergic antibodies. Finally, since we did not include a delay, we could not recreate the phase differences seen in [7], which are an essential difference between POTS and control patients.

The model studied here only includes basic mechanisms necessary to justify the emergence of ∼0.1 Hz oscillations. Future studies should determine if ∼0.1 Hz oscillations differ among phenotypes and test if it is possible to devise a more detailed cell-based model explaining the phenomenon. In addition, our model has the potential to be integrated with models modulating the systems at other frequencies, e.g. respiration, ultradian, circadian or infradian rhythms, and it could be adapted to examine the response in patients exposed to different environments, e.g. high concentrations of carbon monoxide, or an injection of nitric oxide. Future work will contain a more in-depth description of the BR to replicate these phenomena.

The inability of hypovolaemia to cause tachycardia and oscillations may suggest that hypovolaemia is not a distinct POTS phenotype, but it could also indicate that the proposed model needs more details or that we need another approach to model blood loss. Thus effort should be put into exploring the effect of changes in stressed versus unstressed volume or the relationship between BV and CO. Moreover, the model is mechanistic and therefore does not have a parameter specifying some other symptoms that POTS patients experience, such as fatigue, lightheadedness and brain fog. These symptoms can only be incorporated via changes in the control system.

## 5. Conclusion

We have presented a closed-loop differential equation model of the interactions between the BR and cardiovascular system, emphasizing the emergence and amplitude of oscillations in the ∼0.1 Hz frequency range. We have concluded that the HR and peripheral resistance response, represented by Hill coefficients $k_{H}$ and $k_{R}$, respectively, and total BV are critical to the amplitude of low-frequency oscillations while $P_{2H},R_{\mathrm{lpm}}$ and $R_{\mathrm{lpM}}$ are essential to orthostatic tachycardia. Results shared here help explain clinical observations and motivate further modelling and study of POTS to understand better the disease’s pathophysiological aspects and possible treatment options.

## Data Availability

This article has no additional data.
